# Second line therapy with axitinib after only prior sunitinib in metastatic renal cell cancer: Italian multicenter real world SAX study final results

**DOI:** 10.1186/s12967-019-2047-4

**Published:** 2019-08-29

**Authors:** Gaetano Facchini, Sabrina Rossetti, Massimiliano Berretta, Carla Cavaliere, Sarah Scagliarini, Maria Giuseppa Vitale, Chiara Ciccarese, Giuseppe Di Lorenzo, Erica Palesandro, Vincenza Conteduca, Umberto Basso, Emanuele Naglieri, Azzurra Farnesi, Michele Aieta, Nicolò Borsellino, Leonardo La Torre, Gelsomina Iovane, Lucia Bonomi, Donatello Gasparro, Enrico Ricevuto, Michele De Tursi, Rocco De Vivo, Giovanni Lo Re, Francesco Grillone, Paolo Marchetti, Ferdinando De Vita, Claudio Scavelli, Claudio Sini, Salvatore Pisconti, Anna Crispo, Vittorio Gebbia, Antonio Maestri, Luca Galli, Ugo De Giorgi, Roberto Iacovelli, Carlo Buonerba, Giacomo Cartenì, Carmine D’Aniello

**Affiliations:** 1 Departmental Unit of Clinical and Experimental Uro-Andrologic Oncology, Istituto Nazionale Tumori–IRCCS-Fondazione G. Pascale, Via M. Semmola, 80131 Napoli, Italy; 2 Department of Medical Oncology, Centro di Riferimento Oncologico, Istituto Nazionale Tumori CRO, Aviano, PN Italy; 3 UOC of Medical Oncology ASL NA 3 SUD Ospedali Riuniti Area Nolana, Naples, Italy; 4 Division of Oncology, Azienda Ospedaliera di Rilievo Nazionale A. Cardarelli, Naples, Italy; 5 Division of Medical Oncology, Azienda Ospedaliera Universitaria Policlinico di Modena, Modena, Italy; 6 Medical Oncology, Azienda Ospedaliera Universitaria Integrata, University of Verona, Verona, Italy; 7 Department of Clinical Medicine and Surgery, Università degli Studi di Napoli Federico II, Naples, Italy; 8 Division of Medical Oncology, Candiolo Cancer Institute-FPO, IRCCS, Candiolo, Italy; 9 Department of Oncology, IRCCS Istituto Scientifico Romagnolo per lo Studio e la Cura dei Tumori (I.R.S.T.), Meldola, Italy; 10 Medical Oncology Unit 1, Istituto Oncologico Veneto IOV IRCCS, Padua, Italy; 11 Division of Medical Oncology, Istituto Oncologico Giovanni Paolo II, Bari, Italy; 12 University Hospital of Pisa, Oncology Unit 2, Pisa, Pisa, Italy; 13 Medical Oncology Department, National Institute of Cancer, Rionero in Vulture, Italy; 14 Medical Oncology Unit, “Buccheri-La Ferla” Hospital, Palermo, Italy; 15 Medical Oncology Department, “Santa Maria della Scaletta” Hospital AUSL, Imola, Italy; 16 Oncology Department, Ospedale Papa Giovanni XXIII, Bergamo, Italy; 17 Civil Hospital, Parma, Italy; 18 S. Salvatore Hospital, ASL1 Abruzzo, University of L’Aquila, L’Aquila, Italy; 19 Oncology and Experimental Medicine, “G. D’Annunzio” University, Chieti, Italy; 20 Ospedale San Bortolo di Vicenza, Vincenza, Italy; 21 NCI Aviano, Oncology Pordenone-S.Vito, Pordenone, Italy; 22 Medical Oncology Unit Azienda Ospedaliera “Mater Domini”, Catanzaro, Italy; 23 Ospedale Sant’ Andrea Oncology, Roma, Italy; 24 Division of Medical Oncology, University of Campania “L. Vanvitelli”, Napoli, Italy; 25 Medical Oncology Unit, “S. Cuore di Gesù” Hospital, Gallipoli, Italy; 26 Oncologia Medica ASL 2, Olbia, Italy; 27 Medical Oncology Unit, POC SS Annunziata, Taranto, Italy; 28 Medical Oncology Unit, La Maddalena Clinic for Cancer, University of Palermo, Palermo, Italy; 29 Division of Medical Oncology, AORN Dei Colli “Ospedali Monaldi-Cotugno-CTO”, Napoli, Italy

**Keywords:** Axitinib, Sunitinib, Metastatic, Renal cancer, Treatment

## Abstract

**Background:**

This multi-institutional retrospective real life study was conducted in 22 Italian Oncology Centers and evaluated the role of Axitinib in second line treatment in not selected mRCC patients.

**Methods:**

148 mRCC patients were evaluated. According to Heng score 15.5%, 60.1% and 24.4% of patients were at poor risk, intermediate and favorable risk, respectively.

**Results:**

PFS, OS, DCR and ORR were 7.14 months, 15.5 months, 70.6% and 16.6%, respectively. The duration of prior sunitinib treatment correlated with a longer significant mPFS, 8.8 vs 6.3 months, respectively. Axitinib therapy was safe, without grade 4 adverse events. The most frequent toxicities of all grades were: fatigue (50%), hypertension (26%), and hypothyroidism (18%). G3 blood pressure elevation significantly correlated with longer mPFS and mOS compared to G1-G2 or no toxicity. Dose titration (DT) to 7 mg and 10 mg bid was feasible in 24% with no statistically significant differences in mPFS and mOS. The sunitinib-axitinib sequence was safe and effective, the mOS was 41.15 months. At multivariate analysis, gender, DCR to axitinib and to previous sunitinib correlated significantly with PFS; whereas DCR to axitinib, nephrectomy and Heng score independently affected overall survival.

**Conclusions:**

Axitinib was effective and safe in a not selected real life mRCC population.

*Trial registration* INT – Napoli – 11/16 oss. Registered 20 April 2016. http://www.istitutotumori.na.it

## Background

The Target Therapies (TTs) have revolutionized the metastatic Renal Cell Carcinoma (mRCC) treatment with a significant advantage in Overall Survival (OS), from about 9 months in 1995, to a median of 28–29 months in 2013 [1–9]. Axitinib, a selective TKi of VEGFR-1, 2, 3, has been approved in Italy in second line treatment after sunitinib or cytokines failure. The phase III AXIS trials showed a significantly prolonged mPFS with axitinib, 6.7 months vs 4.7 months with sorafenib. In the subgroup of patients, pre-treated with sunitinib, median PFS was 4.8 months with axitinib vs 3.4 months with sorafenib (p = 0.011) [10]. The mOS was 20.1 months with axitinib (95% CI 16.7–23.4) vs 19.2 months with sorafenib (95% CI 17.5–22.3) (HR 0.969, 95% CI 0.800–1.174; p = 0.3744) [11]. Axitinib showed a good safety profile with diarrhea, fatigue and hypertension, as main side effects. At the time of this study analysis, the only registered drugs in this setting were: axitinib, everolimus and sorafenib. To date there are no head-to-head studies or randomized clinical trials, that provide conclusive information about the best second-line. Several ‘real world’ studies confirmed the efficacy and safety of Axitinb in a not selected population [12–24].

## Patients and methods

Our multi-Institutional, retrospective study evaluated the outcomes of mRCC patients all treated in second-line therapy with axitinib after first-line sunitinib failure. Eligible patients were: age ≥ 18 years; histologically confirmed RCC; axitinib for at least 2 months, started between January 2014 and May 2017; at least one radiological assessment (CT scan) of disease (RECIST 1.1 criteria) repeated every 2–3 months; only sunitinib as previous treatment in first line. Axitinib was administered at starting dose of 5 mg bid (10 mg/die). Dose titration (DT) was performed every 2 weeks up to a final step of 10 mg bid in patients without adverse events ≥ grade 2. Primary endpoints were: progression free survival (PFS), overall survival (OS), objective response rate (ORR), disease control rate (DCR), and the safety profile of Axitinib and Sunitinib–Axitinib sequence. ORR was defined as the percentage of partial response (PR) and complete response (CR) during treatment and disease control rate (DCR) as the percentage of PR, CR and stable disease (SD) upon axitinib. Progressive disease (PD) was defined as: radiological tumor progression, or clinical progression, including death. PFS was defined as the interval between the date of the first dose of Axitinib and the date of the disease progression or death from any cause. Overall survival (OS) was defined from the start of axitinib to the date of death from any cause. The secondary objectives included the evaluation of a possible relationship between patients demographic and baseline characteristics, AEs and response to treatment. AEs were graded according to Common Terminology Criteria for Adverse Events (CTCAE version 4.0). Patients demographic and baseline characteristics, treatment patterns and AEs were collected, with categorical variables being described by patients counts and percentages. Univariate analysis for median progression free survival and overall survival was performed by Kaplan–Meier estimator: PFS and OS curves were obtained and selected variables were compared using two-sided log-rank test. Hazard ratios (HR) were calculated by Cox Regression multivariable analysis, performed according to a backward elimination of factors showing a p value ≥ 0.10, and adjusted for age (continuous variable) and center. A p value ≤ 0.05 was considered statistically significant. The SPSS statistical package version 23.0 (SPSS Inc., Chicago, IL) was used for all statistical analysis.

## Results

Between January 2014 and May 2017, twenty-two Italian Oncology Centers collected clinical data regarding 148 patients, after approval by the Institutional Board of National Cancer Institute “G. Pascale”–IRCCS of Napoli, Italy. All patients gave consent to participate. Patients demographic and baseline characteristics were collected in Table 1: median age was 62 years (range: 35–85 years), with good balance between males and females (50.7% vs 49.3%, respectively); 55.4% had ECOG 0 Performance Status. 134/148 (90.5%) patients had undergone prior nephrectomy and only 6% (9/148) had a histological diagnosis other than clear cell carcinoma. Lung was the most affected site of metastases (56.8%) and 22.3% (33/148) of patients had liver metastases. 11.5%, 60.8% and 27.7% patients were MSKCC high risk, intermediate and favorable, respectively otherwise, according to Heng score, 15.5%, 60.1% and 24.4% patients were poor, intermediate and favorable risk, respectively. All patients received sunitinib as first line treatment according to the Italian guidelines: 18% of patients received modified schedule of sunitinib (2 week on 1 week off). All patients started axitinib at standard dose of 5 mg bid. Dose titration to 7 and 10 mg bid was performed in 23.6% of patients. Forty-nine percentage patients received further treatment lines (Table 2).

**Table 1:** SAX patients characteristics

	N = 148	%
Median age years (range)	62	(35–85)
Age		
< 75	126	85%
≥ 75	22	15%
Gender		
Male	75	50.7%
Female	73	49.3%
ECOG PS		
0	82	55.4%
1	61	41.3%
2	5	3.3%
Nephrectomy		
Yes	134	90.5%
No	14	9.5%
MOTZER score		
Poor	17	11.5%
Intermediate	90	60.8%
Favorable	41	27.7%
Heng score		
Poor	23	15.5%
Intermediate	89	60.1%
Favorable	36	24.3%
Principal sites of disease		
Lung	84	56.8%
Lymph node	55	37.2%
Bone	39	26.4%
Liver	33	22.3%
Adrenal glands	10	6.8%
Brain	9	6.1%
Local recurrence	8	5.4%
Pancreas	7	4.7%
Peritoneum	6	4.1%
Contralateral kidney	5	3.4%
Skin	3	2%
Spleen	2	1.4%

**Table 2:** SAX treatments characteristics

	N = 148	(%)
First line		
Sutent	148	100
Sutent schedule		
Modified	27	18
Standard	121	82
Axitinib dose		
Standard	113	76.4
Titration	35	23.6
Therapy after axitinib	73	49

**Fig. 1:**
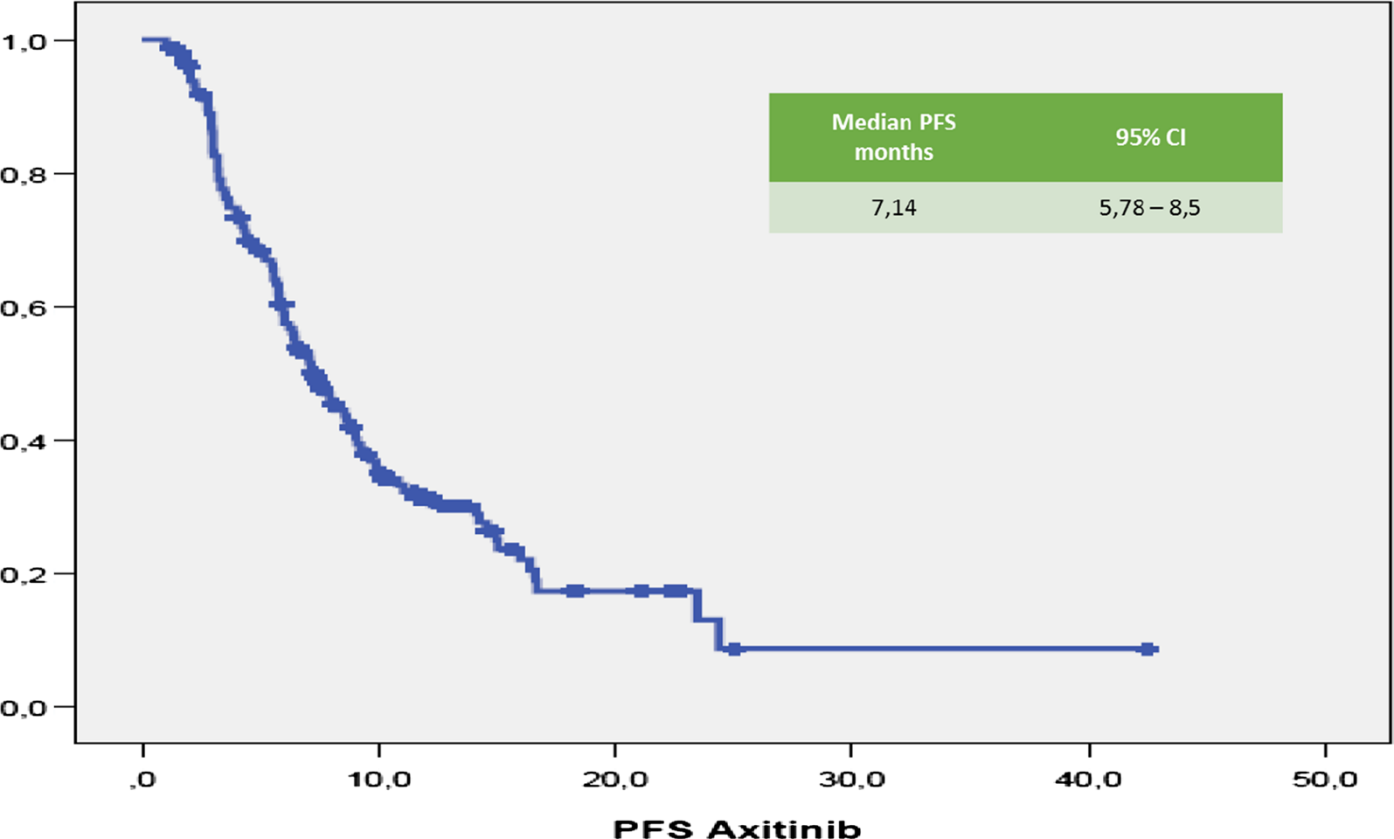
Kaplan-Meier curve of median PFS in our study population

**Fig. 2:**
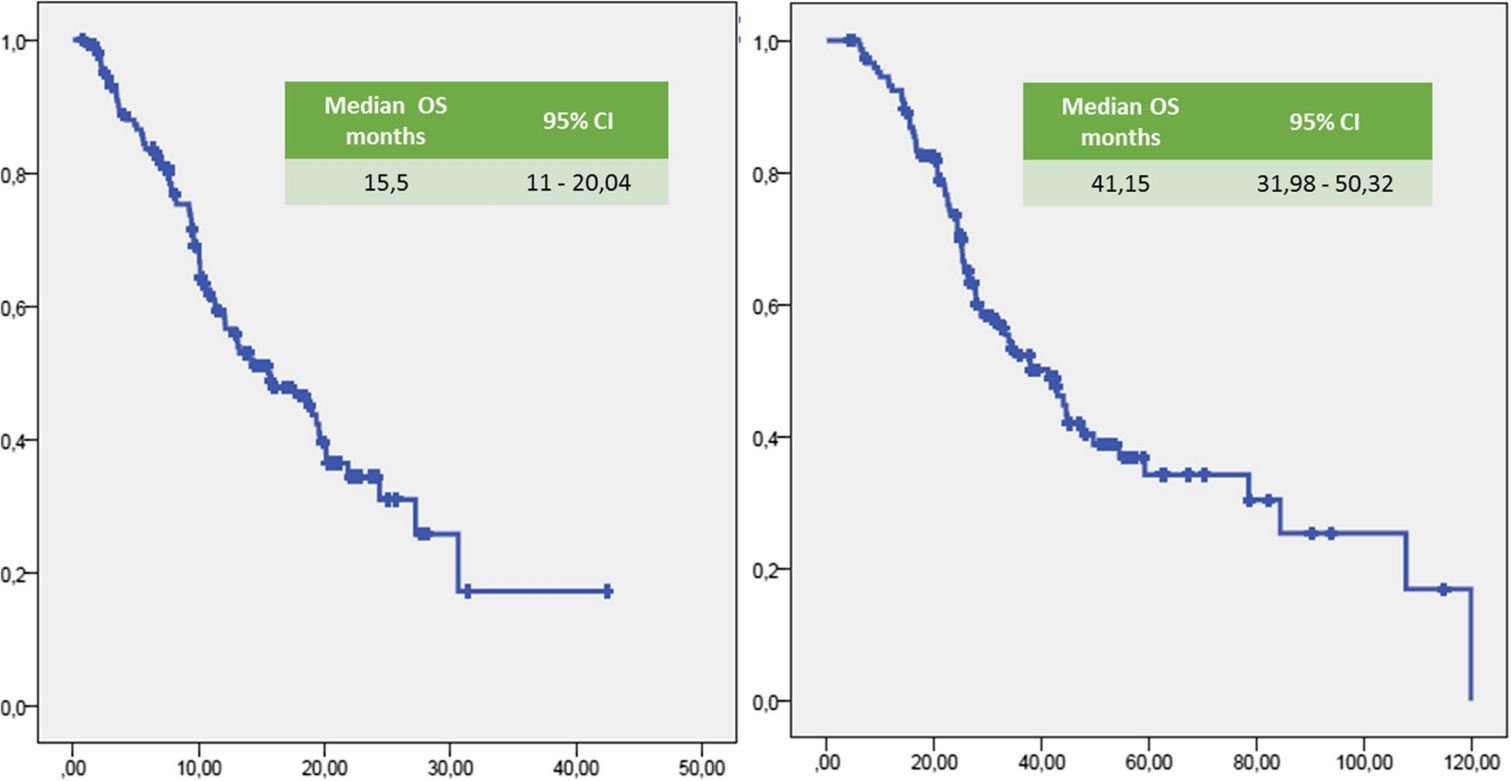
Kaplan-Meier curve of median OS of the patients under study from the start of axitinib

**Fig. 3:**
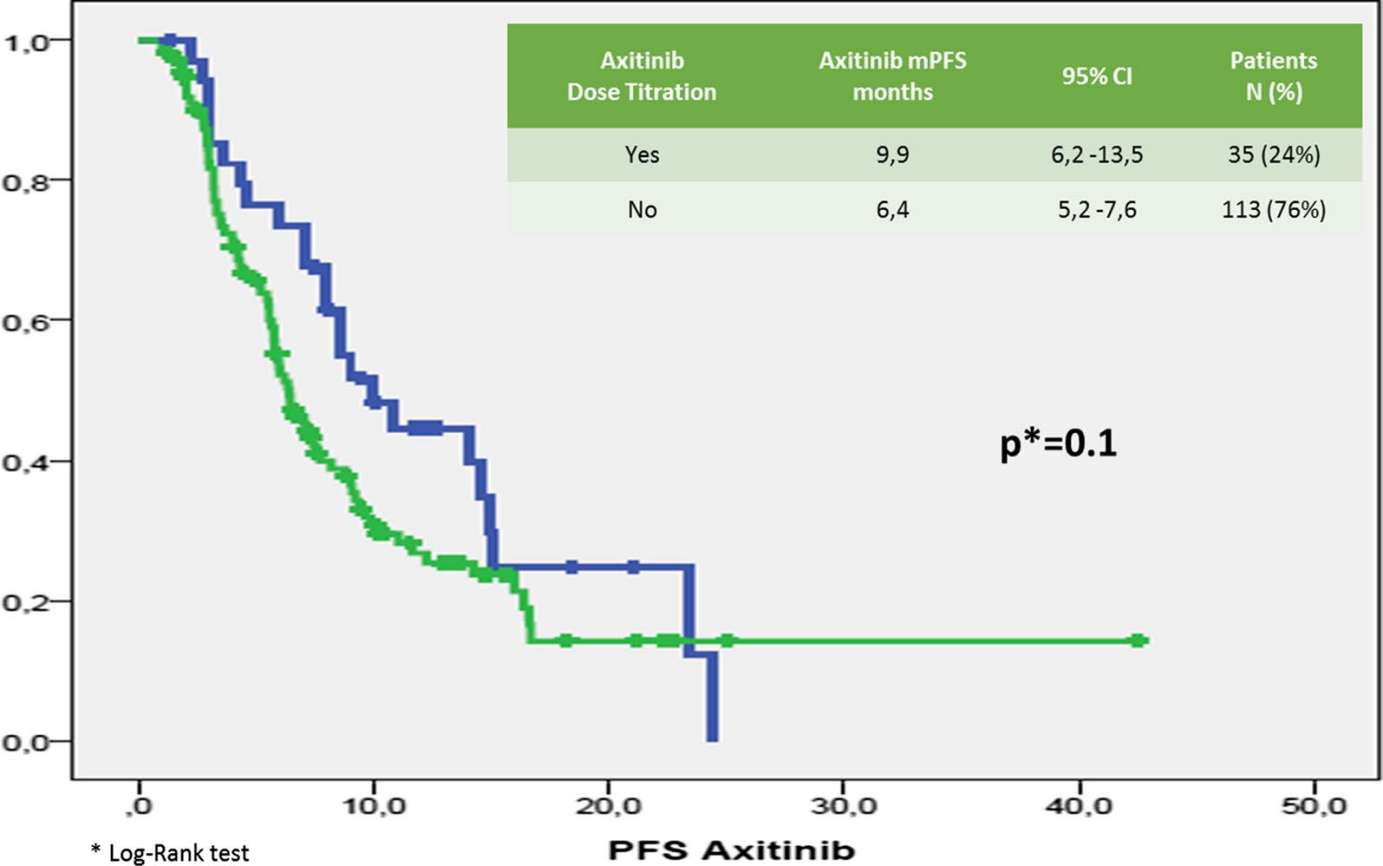
PFS by axitinib dose titration

**Fig. 4:**
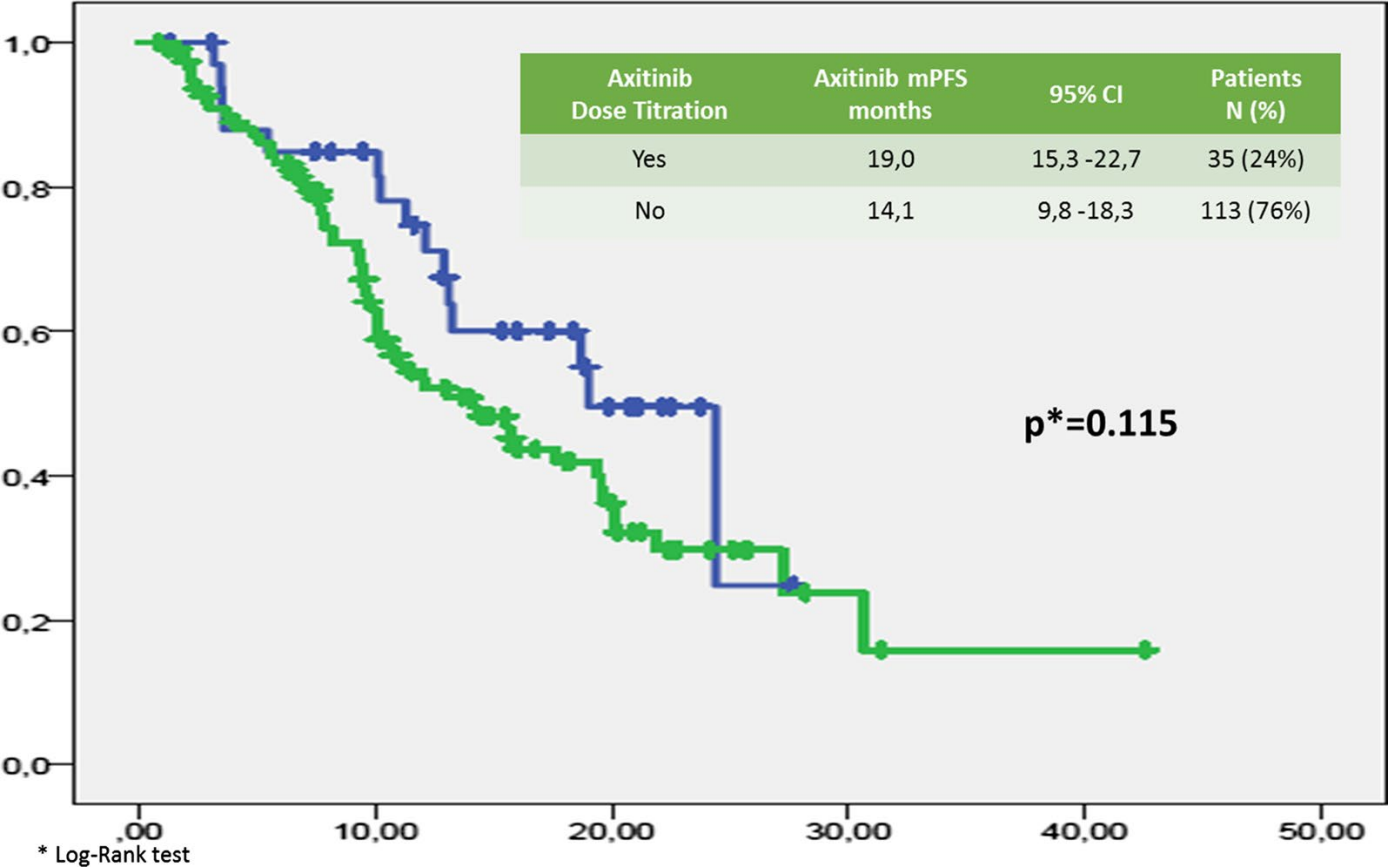
OS by axitinib dose titration

Median (m) PFS was 7.14 months (95% CI 5.78–8.5 months; Fig. 1). Median (m) OS from the start of Axitinib was 15.5 months (95% CI 11–20 months; Fig. 2). The median time of axitinib treatment duration was 8.1 months. The ORR, according to RECIST criteria version 1.1 [25] was 16.6%, with 16% of PR and one patient reached a CR (Table 3) and correlated to a statistically longer (p < 0.0000001) mPFS, 15.5 months (95% CI 7.9–22.1 months) vs 3.2 months (95% CI 2.95–3.445 months), respectively. The DCR with Axitinib was 70.6% and correlated to a statistically longer (p < 0.0000001) mPFS, 9.9 months (95% CI 7.59–12.22 months) vs 3.2 months (95% CI 2.95–3.44 months), respectively. mOS according to DCR and ORR upon axitinib was 20.1 vs 7.83 months (p < 0. 0000001) and 27.2 vs 7.8 months (p = 0. 000026), respectively. DCR and ORR to previous Sunitinib treatment were associated with longer statistically mPFS, 7.96 months (95% CI 6.49–9.42 months, p = 0. 00031) and 7.7 months (95% CI 5.8–9.7 months, p = 0.0011) vs 4.0 months (95% CI 1.14–6.68 months) and 4.0 months (95% CI 1.4–6.7 months), respectively; no statistically significant differences in mOS according to DCR upon sunitinib was recorded, 17.6 months (95% CI 12.9–22.4 months, p = 0.094) vs 7.8 months (95% CI 4.9–10.8 months); conversely, patients who achieved ORR with first line sunitinib had a significant longer median OS, 19.0 months (95% CI 12.7–25.4 months, p = 0.049) vs 4.0 months (95% CI 4.9–10.7 months). With stratifying patients by duration of prior sunitinib therapy (≤ vs > median duration), a statistically significant difference in mPFS was reported: patients with a median duration of Sunitinib ≥ 13.1 months experienced disease progression upon axitinib later than ones who progressed within 13 months (8.8 months vs 6.3 months, p = 0.021), without any difference in mOS (p = 0.151). We reported no differences in terms of mPFS according to previous sunitinib administration schedule, 13.1 months (95% CI 11.7–14.6 months) vs 12.7 (95% CI 9.7–15.7 months) (standard schedule vs modified schedule; p = 0.096); no difference in mOS (p = 0.205) according to alternative schedule vs standard, 17.6 months (95% CI 12.6–22.7 months) vs 10.2 months (95% CI 8.7–11.7 months). When patients were stratified by Heng score, mPFS was 5.8, 7.0 and 9.0 months according to poor, intermediate and favorable risk group (p = 0.066), with statistically significant difference in mOS (9.4 vs 14.3 vs 20.1 months, respectively p = 0.002); similar results were obtained by using Motzer score. Patients with better ECOG PS (0) experienced longer mPFS, 9.08 months (95% CI 6.80–11.3 months, p = 0.026) vs 6.2 months (95% CI 5.5–6.9 months) and mOS, 27.2 months (95% CI 12.0–42.4 months, p = 0.003) vs 10.9 months (95% CI 8.3–13.6 months). Prior nephrectomy significantly correlated to a longer mPFS, 7.7 vs 4.4 months (p = 0.001), as well as to longer mOS, 18.7 vs 8.2 months, (p = 0.000004). Axitinib at standard schedule of 5 mg bid was safe without grade 4 toxicity. Dose reduction occurred in 24% (35/148): the most common adverse events of all grades were fatigue (50.7%), gastro-intestinal disorders (36.5%), hypertension (26.4%), hypothyroidism (18.2%), dysphonia (12.2%), hand-foot syndrome (14.2%) (Table 5). At univariate analysis G3 blood pressure elevation (systolic ≥ 160 mmHg and/or diastolic ≥ 100 mmHg) significantly correlated with longer mPFS and mOS compared to G1–G2 or no toxicity (mean PFS 28.8 months, p = 0.017—mean 6 OS 38.15 months, p = 0.017—median survival times not reached for both analysis). Noteworthy, men compared to women showed both a longer mPFS (9 vs 5.8 months, p = 0.014) and mOS (19.5 vs 12 months, p = 0.048). The Sunitinib–Axitinib sequence, was well-tolerated, without worsening in side effects, particularly in terms of hypertension and hand–foot syndrome, with a mOS of 41.15 months (95% CI 32–50.32 months; Fig. 2). Tables 5 and 6 summarized the adjusted hazard ratios (HR) for PFS and OS: the Cox multivariate model, performed according to a backward elimination of factors showing a p value ≥ 0.10, was then adjusted for age, gender, and center; gender (male vs female: HR 0.567, 95% CI 0.378–0.851, p value = 0. 006), DCR upon axitinib (HR 0.171, 95% CI 0.107–0.272, p value < 0.0000001) and upon prior sunitinib (HR 0.549, 95% CI 0.308–0.977, p value = 0.04) showed a significant independent impact in terms of PFS; on the other hand, DCR upon axitinib (HR 0.336, 95% CI 0.192–0.590, p value = 0.0001), Heng score (poor prognosis vs favorable prognosis: HR 3.4, 95% CI 1.374–8.541, p value = 0.008—intermediate prognosis vs favorable prognosis: HR 2.06, 95% CI 1.04–4.0, p value = 0.04) and prior nephrectomy (HR 0.319, 95% CI 0.153–0.664, p value = 0.0022) independently affected overall survival (Table 4). Dose escalation to 7 or 10 mg bid was feasible in 35/148 patients (24.2%). mPFS was longer, but not statistically significant, than patients without dose titration, 9.9 months (95% CI 6.2–13.5 months, p = 0.1) vs. 6.4 months (95% CI 5.2–7.6 months), respectively. No difference in mOS was observed too (p = 0.115, Figs. 3, 4). Dose titration was well-tolerated without significant increase in side effects (Tables 5, 6, 7).

**Table 3:** Objective response in our study population

	Patient n = 148
Best response, (%)	
CR	0.6
PR	16
SD	54
PD	29.4
DCR (CR + PR + SD)	70.6
ORR (CR + PR)	16.6

**Table 4:** Univariate analysis of PFS and OS in our study population

	p value	
	mPFS	mOS
Tumor response rate to axitinib		
DCR	< 0.0000001	< 0.0000001
ORR	< 0.0000001	0.000026
Tumor response rate to prior sunitinib		
DRC	0.00031	0.094
ORR	0.0011	0.049
Duration prior sunitinib treatment ≥ 13.1 vs < 13.1 mo	0.21	0.151
HENG score	0.066	0.002
ECOG PS	0.026	0.003
Prior nephrectomy	0.001	0.000004
G3 blood pressure	0.017	0.017

**Table 5:** Axitinib toxicity

Adverse event (%)	Standard dose			Titration		
	Grade 1–2 87.3%	Grade 3 12.7%	Grade 4 0%	Grade 1–2 86.6%	Grade 3 13.4%	Grade 4 0%
Haematologic	9.5	0.7	–	11.4	2	–
Hypertension	20.9	5.4	–	25.7	5.7	–
Gastro-intestinal	32.4	4.1	–	34.3	–	–
Hypothyroidism	17.6	0.7	–	25.7	–	–
Stomatitis/mucositis	8.1	–	–	8.5	–	–
Fatigue	43.2	7.4	–	48.6	8.6	–
Hepatic	2.8	2	–	2.9	5.7	–
Hand-foot syndrome	12.2	2	–	17.2	2.9	–
Dysphonia	11.5	0.7	–	11.4	2.9	–

**Table 6:** Cox multivariate analysis for PFS

	Progression-free survival (PFS)		
	HR	(95% CI)	*p* value
DCR axitinib	0.171	(0.107–0.272)	< 0.0000001
DCR sunitinib	0.549	(0.308–0.977)	0.041
Heng score			
Good prognosis	1		0.174
Poor prognosis	1.909	(0.964–3.779)	0.064
Intermediate	1.249	(0.752–2.073)	0.391
Nephrectomy			
Yes	0.572	(0.305–1.072)	0.081
Gender			
Male	0.567	(0.378–0.851)	0.006

**Table 7:** Cox multivariate analysis for OS

	Overall survival (OS)		
	HR	(95% CI)	p-value
DCR axitinib	0.336	(0.192–0.590)	0.00015
Performance status			
ECOG 0	1		0.058
ECOG 1	0.872	(0.183–4.160)	0.863
ECOG 2	1.706	(0.359–8.108)	0.502
Heng score			
Good	1		0.025
Poor	3.426	(1.374–8.541)	0.008
Intermediate	2.057	(1.040–4.068)	0.038
Nephrectomy			
Yes	0.319	(0.153–0.664)	0.002
Istology			
Clear cell carcinoma	0.402	(0.149–1.079)	0.070
Gender			
Male	0.629	(0.371–1.066)	0.085

## Discussion

Currently the goal of mRCC treatment strategy is represented by the correct use of the approved drugs in a sequential algorithm [26, 27]. Axitinib is licensed in Italy for the treatment of mRCC patients only after failure of sunitinib or cytokines therapy. We report herein the retrospective data of axitinib in Italian real-life practice for mRCC: despite our population was more “battered” than the one investigated in AXIS trial, our results are consistent with AXIS ones, confirming the efficacy of axitinib in second line treatment [10, 11], with ORR, mPFS and mOS of 16.6%, 7.14 and 15.5 months, respectively. Fifteen percentage of our study population was over 75 years, normally under-represented in clinical trials [28]. The elderly patients are usually a frail population with a lower performance status (PS), poor tolerance to medical treatments and multiple co-morbidities [29]. To date few data are available concerning the use of axitinib in elderly mRCC patients [30–32]. Our results showed no differences in both mPFS [6.4 months (95% CI 4. 95–7.95, p = 0.74)] and mOS [13.0 months (95% CI 5.9–20.15, p = 0.72)] than younger patients. In addition, there was no significant difference in the incidence of AEs or dose reduction, or discontinuation. The efficacy and safety of the VEGF-TKI -VEGF-TKI treatment sequence has been confirmed by various trials, showing a statistically longer mPFS and in some of these mOS too [10, 11, 26, 33, 34]. Leung et al. indicated axitinib as more appropriate TTs option, compared to sorafenib and pazopanib, in the second line setting; in particular, axitinib is associated with the lowest risk of withdrawal due to adverse events [35]. In post hoc analysis of the AXIS trial, Escudier et al. evaluated the efficacy of axitinib by response and duration of prior sunitinib or cytokines treatment, showed no statistically significant differences in PFS or OS in responders vs non-responders, although a significantly longer PFS and OS was reported in patients who had received a longer prior cytokines treatment [36]. On the contrary, our analysis showed that longer previous sunitinib duration (≤ vs > median duration), correlated with a statistically significant difference in mPFS (8.8 vs 6.3 months, p = 0.021), without any difference in mOS (p = 0.151). The same conclusion was reached by Elaidi et al. who showed that patients who remained on first-line TKI treatment between 11 and 22 months benefited from a TKI rechallenge rather than from second-line mTORi (PFS: 9.4 vs 3.9 months, p = 0.003) [37]. Higher ORR (20–30%) was reported with VEGF-TKI compared to mTORi (≤ 10%), which is supported by our analysis [38]. Dose titration to 7 or 10 mg bid was feasible in 24% (35/148) of our patients, lower than the axitinib Asian trial (61.5%) [39] or the AXIS trial (37%) [10], but higher than other real-world studies (16%) [21–23, 40, 41]. We reported no differences in both mOS (p = 0.115) and mPFS (p = 0.1), in accordance to the phase II study of first-line axitinib [17, 23] but in contrast to Matias et al. results, in which dose escalation at 2-weeks was associated to better ORR, PFS and TTF, but not OS. Patients with better ECOG PS (0) experienced longer mPFS, 9.08 (p = 0.026) vs 6.2 months and mOS, 27.2 (p = 0.003) vs 10.9 months. Prior nephrectomy significantly correlated with longer mPFS, 7.7 vs 4.4 months (p = 0.001), as well as longer mOS, 18.7 vs 8.2 months, (p = 0.000004). Axitinib at standard dose of 5 mg bid was safe, a dose reduction occurred in 24% (35/148), without any case of discontinuation: the most common AEs of all grades were: fatigue (50.7%), gastro-intestinal disorders (36.5%), hypertension (26.4%), hypothyroidism (18.2%), dysphonia (12.2%), hand-foot syndrome (14.2%) (Table 5). Our data showed a lower incidence of AEs than AXIS trial, the higher incidence of fatigue in our experience, was probably due to the difficulty to distinguish and explain to the patients the difference between fatigue and asthenia. All these results suggest that axitinib treatment is feasible and safe in this unselected real-world population. At univariate analysis hypertension G3 blood pressure elevation (systolic ≥ 160 mmHg and/or diastolic ≥ 100 mmHg) significantly correlated with longer mPFS and mOS compared to G1-G2 or no toxicity (mean PFS 28.8 months, p = 0.017—mean OS 38.15 months, p = 0.017—median survival times not reached for both analysis Table 6, 7). Our data are consistent with other real-world studies [42, 43] and AXIS trial, suggesting that the development of hypertension during the treatment could be a surrogate of survival in this population. It was interesting to note that the 18% (27/148) of patients enrolled in our study, adopted a modified schedule of sunitinib in first line (2 weeks on 1 week off), without showing any difference in outcomes. These data confirm those of others retrospective studies that evaluated sunitinib alternative schedules, showing a reduction in the AEs and achieving comparable outcomes to the standard schedule [44–46]. The identification of effective prognostic factors in mRCC patients receiving axitinib represents a new challenge. In these series we identified the following independent prognostic indicators: gender (male), DCR upon axitinib and prior sunitinib for PFS, and DCR upon axitinib, Heng score (poor prognosis vs intermediate vs good prognosis) and prior nephrectomy for OS. The sequence TKI–TKI (sunitinib-axitinib) was well tolerated without worsening in side effects, the global mOS was 41.15 months, higher than AXIS trial (33.7 months). The main limitation of our analysis was represented by the small patient numbers, selection bias, the retrospective nature, without centralized data review. Recently the results of three major clinical trials involving nivolumab, cabozantinib, and lenvatinib plus everolimus, showed superior efficacy in terms of response rates (RR) and OS in second-line setting [47–50] and these will change dramatically the therapeutic sequence in second-line setting. To date, there are few data about the best sequential therapeutic algorithm beyond first-line VEGF TKIs, and no head-to-head study between these new drugs and the currently approved agents are ongoing [51–54]. The mTORi everolimus is the only drug tested head-to-head with nivolumab, cabozantinib and lenvatinib plus everolimus, and no data are available with axitinib as comparator. Treatment selection in second line-setting, is based on several factors, including patient health status, contraindications and comorbidities, histologic RCC subtype, safety profiles, and previous treatment. Recently, Bracarda et al. published a Prognostic Factor Analyses from the AXIS Trial, that as well as our data, identified a subgroup of patients who had a long-term benefit with axitinib treatment. Therefore, axitinib could be suitable (post sunitinib) 2nd line treatment option for mRCC selected patients with VEGF-dependent mRCC, favourable/intermediate risk, low tumour burden, and no bone or liver metastases and with long life expectancy [55]. In the new era of Immunotherapy, are VEGF-TKIs still a valid option for mRCC treatment? The angiogenesis plays a central role in the RCC tumorigenesis and immunogenicity. The prevalence of pro-angiogenic factors over anti-angiogenic signals promotes an immunosuppressive tumor microenvironment, through abnormal tumor vessel formation and dysregulation of various immune cells. Therefore, anti-angiogenic therapy remains the gold standard in selected patients (VEGF-dependent favourable mRCC in all setting) and increases the efficacy of immunotherapy, modulating immune responses, increasing anticancer immune-trafficking and activity, through the regulation of tumor vessels and reducing suppressing cytokines and infiltrating T regs [54, 56, 57]. Different phase 3 trials evaluated or are evaluating combination of immune checkpoint inhibitors, such as anti PD-1 nivolumab and anti CTLA-4 ipilimumab, or anti PD-1/PDL-1 and VEGFR-TKI in first-line treatment, with impressive results that will dramatically impact on the choice of the first and second-line treatments (Table 8).

**Table 8:** Real world trial data comparison

	mPFS (mo)	mOS (mo)	DCR (CR + PR + SD) (%)
SAX real world	7.14	15.5	70.6
Spanish real world	4.4	10.8	65.7
France real world	8.3,	16.4,	72
AXIS	6.5	15.2	69.3

## Conclusions

Evidences emerging from our retrospective analysis are consistent with the available literature and confirm the efficacy and safety of axitinib in a not selected population, particularly in patients who most benefited from first-line sunitinib (VEGF-dependent mRCC). The advent of new drugs such as nivolumab and cabozantinib has further improved the therapeutic landscape of second line setting. Prospective trial will be needed to assess the right sequence of anti PD-1/PD-L1 and VEGF/VEGFRi and moreover, head to head studies will be needful to determine the best VEGFRi (cabozantinib vs axitinib) in second line setting, mostly after the impressive results of the combination trials of immune checkpoint inhibitors and immune checkpoint inhibitors with VEGFR-TKIs, in first-line therapy.

## Data Availability

The datasets during and/or analysed during the current study available from the corresponding author on reasonable request.
